# Beyond a Symptom: A Mechanism-Based Multidisciplinary Framework for the Diagnosis and Management of Chronic Cough

**DOI:** 10.3390/diagnostics16213701

**Published:** 2026-11-09

**Authors:** Engin Dursun, Emine Elif Altuntaş, Haldun Oğuz, Selmin Karataylı Özgürsoy, Esma Altan, Saime Güzelsoy Sağıroğlu, Yusuf Aydemir, Güleser Saylam, Adile Berna Dursun, Celil Göçer

**Affiliations:** 1 Department of Otorhinolaryngology, Faculty of Medicine, Lokman Hekim University, 06510 Ankara, Türkiyeguleser.saylam@lokmanhekim.edu.tr (G.S.);; 2 Department of Otorhinolaryngology, Faculty of Medicine, Ufuk University, 06520 Ankara, Türkiye; 3 Department of Otorhinolaryngology, Ankara Etlik City Hospital, Ministry of Health, 06170 Ankara, Türkiye; 4 Department of Otorhinolaryngology, HG Hospital, 46050 Kahramanmaraş, Türkiye; 5 Department of Pulmonology, Faculty of Medicine, Sakarya University, 54290 Sakarya, Türkiye; 6 Department of Pulmonology/Allergy and Immunology, Faculty of Medicine, TOBB University of Economics and Technology, 06560 Ankara, Türkiye; aberna.dursun@gmail.com

**Keywords:** chronic cough, cough hypersensitivity syndrome, refractory chronic cough, unexplained chronic cough, treatable traits, laryngeal hypersensitivity, behavioral cough suppression therapy, objective cough monitoring, multidisciplinary care, precision medicine

## Abstract

Chronic cough may persist despite appropriate treatment of associated conditions, creating uncertainty about the relevance of coexisting abnormalities and therapeutic priorities. This evidence-informed narrative review draws on searches of PubMed/MEDLINE, Scopus, and Web of Science and relevant guidelines to propose a mechanism-based multidisciplinary framework for adults, particularly those with refractory or unexplained chronic cough. The nine-step pathway integrates established disease assessment, characterization of cough hypersensitivity and laryngeal involvement, provisional weighting of treatable traits and modifiable contributors, targeted multidisciplinary input, individualized intervention, and reassessment. Within this proposed framework, findings are considered dominant, contributory, incidental, or currently inactive according to converging clinical evidence; insufficiently evaluated findings remain uncertain. Treatment response is interpreted by distinguishing improvement in the targeted trait from improvement in cough. Discordant or inadequate responses prompt reassessment of treatment adequacy and mechanistic relevance. Patient-reported outcomes, objective cough monitoring when available, and trait-specific measures inform treatment evaluation and subsequent re-phenotyping. The weighting categories and integrated pathway represent the authors’ conceptual synthesis and have not been prospectively validated. Their reproducibility, feasibility, clinical effectiveness, and cost-effectiveness require evaluation.

## 1. Introduction

Chronic cough is among the most common reasons for seeking medical attention worldwide and remains a major source of morbidity, healthcare utilization, and reduced quality-of-life (QoL). In adults, chronic cough is generally defined as cough persisting for more than 8 weeks [1,2,3]. Although traditionally regarded as a symptom secondary to lower-airway or upper airway disease, contemporary evidence increasingly suggests that chronic cough is a complex clinical syndrome with distinct pathophysiological mechanisms that extend beyond the simple manifestations of an underlying disease [4,5,6,7].

Guideline-based evaluation and treatment of associated conditions remain essential, but cough may persist despite appropriate management [6,8,9]. The diagnostic challenge therefore extends beyond identifying an associated disease to determining which mechanisms and treatable traits remain clinically relevant to cough persistence.

Existing reviews have examined cough hypersensitivity, its neural mechanisms, and therapeutic implications [10,11]. Multidisciplinary frameworks have also been proposed for unexplained chronic aerodigestive symptoms [12].

Building on this evidence, we propose a chronic cough-specific conceptual framework that links the estimated clinical relevance of coexisting treatable traits and modifiable contributors to therapeutic prioritization, targeted multidisciplinary input, and reassessment. Its emphasis is on making the reasoning process explicit, including how discordance between improvement in a targeted trait and improvement in cough should prompt reconsideration of the working mechanistic interpretation. The proposed weighting categories and nine-step pathway represent the authors’ synthesis and require prospective validation.

## 2. Literature Search and Evidence Synthesis

This evidence-informed narrative review was developed through a structured literature search of PubMed/MEDLINE, Scopus, and Web of Science, supplemented by searches of relevant international guidelines and consensus statements, covering literature available until August 2026. Search terms included combinations of “chronic cough,” “refractory chronic cough,” “unexplained chronic cough,” “cough hypersensitivity syndrome,” “treatable traits,” “diagnostic assessment,” “phenotyping,” “biomarkers,” “laryngeal hypersensitivity,” “laryngeal dysfunction,” “upper airway cough syndrome,” “gastroesophageal reflux,” “behavioral cough suppression therapy,” “speech-language pathology,” “neuromodulators,” “P2X3 receptor antagonists,” “objective cough monitoring,” “digital cough analysis,” “artificial intelligence,” and “multidisciplinary cough clinic.” Targeted supplementary searches and source checks addressed questionnaire development, clinical phenotyping and endotyping, pharmacological mechanisms, and relevant therapeutic updates.

International guidelines, consensus statements, systematic reviews, randomized controlled trials, mechanistic reviews, and clinically relevant observational studies published in English were prioritized. The review focused primarily on chronic cough in adults, including relevant clinical subpopulations, with studies selected for their relevance to mechanisms, diagnostic phenotyping, multidisciplinary management, and therapeutic decision-making. When multiple publications addressed the same question, greater weight was given to more contemporary and methodologically robust evidence.

Because the purpose of this article was to develop a conceptual, mechanism-based, multidisciplinary framework rather than to conduct a formal systematic review or meta-analysis, the evidence was synthesized narratively, rather than quantitatively. Evidence was interpreted with particular attention to clinical applicability, consistency with contemporary guidelines, mechanistic relevance, and potential contributions to multidisciplinary diagnostic and therapeutic decision-making. The individual diagnostic and therapeutic components were drawn from the literature, whereas their organization into the proposed nine-step pathway and mechanistic weighting categories represents the authors’ conceptual synthesis. The integrated framework was not empirically tested or validated in this review. OpenAI ChatGPT was used to assist with drafting and language refinement, manuscript organization, identification of relevant literature and review of source–text consistency, and development of conceptual graphical materials.

## 3. Epidemiology and Clinical Burden

Chronic cough is estimated to affect approximately 5–10% of adults, with prevalence varying by geographical setting and case definition [2,13]. Middle-aged and older women are overrepresented in many specialist chronic cough cohorts, although female predominance is not consistently observed in population-based studies [14,15]. In addition to its prevalence, chronic cough has substantial physical, psychological, social, and economic consequences. Patients frequently report sleep disturbances, urinary incontinence, musculoskeletal pain, social embarrassment, impaired work productivity, anxiety, depression, and persistent fear of serious diseases, all of which contribute to significant reductions in health-related QoL [6,13,16,17,18].

## 4. From Etiology to Refractory and Unexplained Chronic Cough

Clinical management has traditionally focused on identifying and treating the underlying cause, most commonly asthma, gastroesophageal reflux disease (GERD), or upper airway cough syndrome [1,2,3].

Refractory chronic cough (RCC) refers to cough that persists despite systematic evaluation and adequate treatment of identified cough-associated conditions or treatable traits [2,19]. Unexplained chronic cough (UCC) refers to cough that remains unexplained after guideline-based investigation and supervised trials of clinically indicated treatments [20].

Cough hypersensitivity syndrome (CHS) provides a complementary framework for understanding persistent symptoms in patients with RCC or UCC [10,21].

The key concepts underpinning this evolving framework are presented in Table 1.

## 5. Mechanistic Basis of Chronic Cough

Originally proposed by Morice et al., CHS describes a clinical condition characterized by heightened cough reflex responsiveness to stimuli that would not normally provoke coughing in healthy individuals [21]. Patients frequently report coughing in response to innocuous triggers such as talking, laughing, perfumes, cold air, eating, temperature changes, or exposure to environmental irritants. Many patients also describe abnormal laryngeal sensations, such as throat irritation, tickling, dryness, or an irresistible urge to cough, preceding cough episodes [21,22]. These clinical features suggest that persistent cough cannot be explained solely by structural airway abnormalities or ongoing inflammation [10,21].

Nevertheless, CHS remains a clinical construct without a universally accepted objective diagnostic gold standard. Symptoms such as allotussia, hypertussia, and an exaggerated urge to cough do not, by themselves, identify a specific peripheral or central neural abnormality. In this review, sensory-neural sensitization describes increased responsiveness within cough pathways; it does not, by itself, establish a diagnosis of neuropathy [4,11]. This concept is particularly useful in adults with persistent dry or minimally productive cough, including appropriately evaluated patients with RCC or UCC, but should complement rather than replace investigation and treatment of underlying disease. Productive cough, suspected infection, structural airway disease, aspiration, or red-flag features require cause-directed assessment and management. Cough-provocation tests may characterize sensory responsiveness in research, but they should not be interpreted as stand-alone diagnostic tests for RCC or as proof of a specific mechanistic endotype [2,23].

Current evidence indicates that cough hypersensitivity arises from complex interactions between the peripheral and central neural mechanisms [10,24]. Peripheral sensitization involves increased excitability of vagal afferent sensory pathways and altered signaling through sensory receptors and ion channels, including transient receptor potential vanilloid-1 (TRPV1), transient receptor potential ankyrin-1 (TRPA1), and P2X purinoceptor 3 (P2X3) receptors [4,24]. Repeated inflammatory, infectious, reflux-related, or mechanical injury may induce long-lasting neuroplastic changes in these pathways. Simultaneously, central sensitization appears to amplify sensory processing within the brainstem and higher cortical centers, lowering the threshold for cough generation and perpetuating symptoms even after the initial trigger has been resolved [4,10]. Functional MRI findings suggest that altered central processing in chronic cough is region-specific rather than a uniform increase in neural activation. In the fMRI analysis of 28 participants with chronic cough and 28 controls at equivalent urge-to-cough levels, patients showed reduced activation in medullary regions and increased activation in a midbrain region, whereas activation in cortical regions encoding cough sensations did not differ significantly between groups. These findings support altered central processing but do not establish a single neural mechanism applicable to all patients with cough hypersensitivity [25].

Experimental evidence suggests that cough hypersensitivity may develop without overt airway inflammation. In a guinea pig model, prolonged nitric oxide (NO) exposure has been shown to induce cough hypersensitivity through activation of the hypoxia-inducible factor 1α (HIF1α)–TRPV1 pathway without significant inflammatory airway changes, supporting non-inflammatory neural sensitization as a potential mechanism of chronic cough [26].

Cough hypersensitivity has conceptual parallels with chronic pain, itch, and other sensory hypersensitivity syndromes [10,27]. 

Originally developed for chronic airway diseases, the treatable traits approach seeks to identify specific factors that contribute to symptom generation and can be directly targeted by therapy [28]. In chronic cough, these factors may include eosinophilic airway inflammation, upper airway disease, reflux-related sensory activation, laryngeal hypersensitivity, dysfunctional breathing, cough hypersensitivity, behavioral contributors, obesity, smoking-related airway injury, sleep-disordered breathing, and psychological comorbidities that may influence symptom perception, coping, and overall symptom burden [6,23,29].

In a multicenter Korean Chronic Cough Registry involving 426 adults with chronic cough, cough hypersensitivity symptoms were independently associated with cough persistence, whereas several traditionally recognized traits, including type 2 inflammation, chronic rhinosinusitis (CRS), gastroesophageal reflux-related symptoms, airflow obstruction, and obesity, were not independently associated with cough persistence. These observational findings support cough hypersensitivity symptoms as markers of persistence, but do not establish causal dominance or imply that the other evaluated traits are unimportant treatment targets [30]. 

Oliveira et al. proposed reclassifying chronic cough according to identifiable treatable traits [31]. RCC and UCC are retained in this review to describe the patient populations defined by these terms in the supporting literature.

Eosinophilic airway inflammation may represent an actionable inflammatory trait, whereas upper airway disease and reflux-related events may contribute through inflammatory, mechanical, or sensory pathways [2,17,32,33,34,35,36]. The larynx may function as a sensorimotor interface in which afferent hypersensitivity, protective airway reflexes, phonatory behavior, inducible laryngeal obstruction, and breathing-pattern abnormalities converge [22,37,38]. Reflux may contribute through microaspiration, vagally mediated esophagobronchial reflexes, non-acid reflux, esophageal dysmotility, or sensitization of laryngeal and airway sensory pathways. Bidirectional cough–reflux interactions may further perpetuate symptoms, whereby reflux facilitates cough through sensory activation, whereas repeated coughing promotes additional reflux events [2,33,35]. Peripheral and central neural sensitization may further amplify cough responsiveness, while psychological and behavioral factors may modify symptom perception, coping, and overall symptom burden without necessarily representing the primary cause of cough [4,39,40]. Psychological distress may be a consequence as well as a modifier of persistent cough and should not, on its own, justify psychogenic attribution [39].

Importantly, the presence of an abnormality does not by itself establish its causal relevance to cough. Nonspecific laryngeal findings, evidence of reflux, inflammatory abnormalities, or other coexisting conditions should therefore be interpreted within the overall clinical phenotype and in relation to other potentially active mechanisms [2,12,33,35,41]. Multiple mechanisms may coexist within the same patient, and their relative contribution to cough persistence may vary over time.

Although these mechanisms are presented here within an integrated rather than specialty-specific framework, different clinical disciplines provide complementary expertise in their assessment and management, as summarized in Table 2.

**Figure 1:**
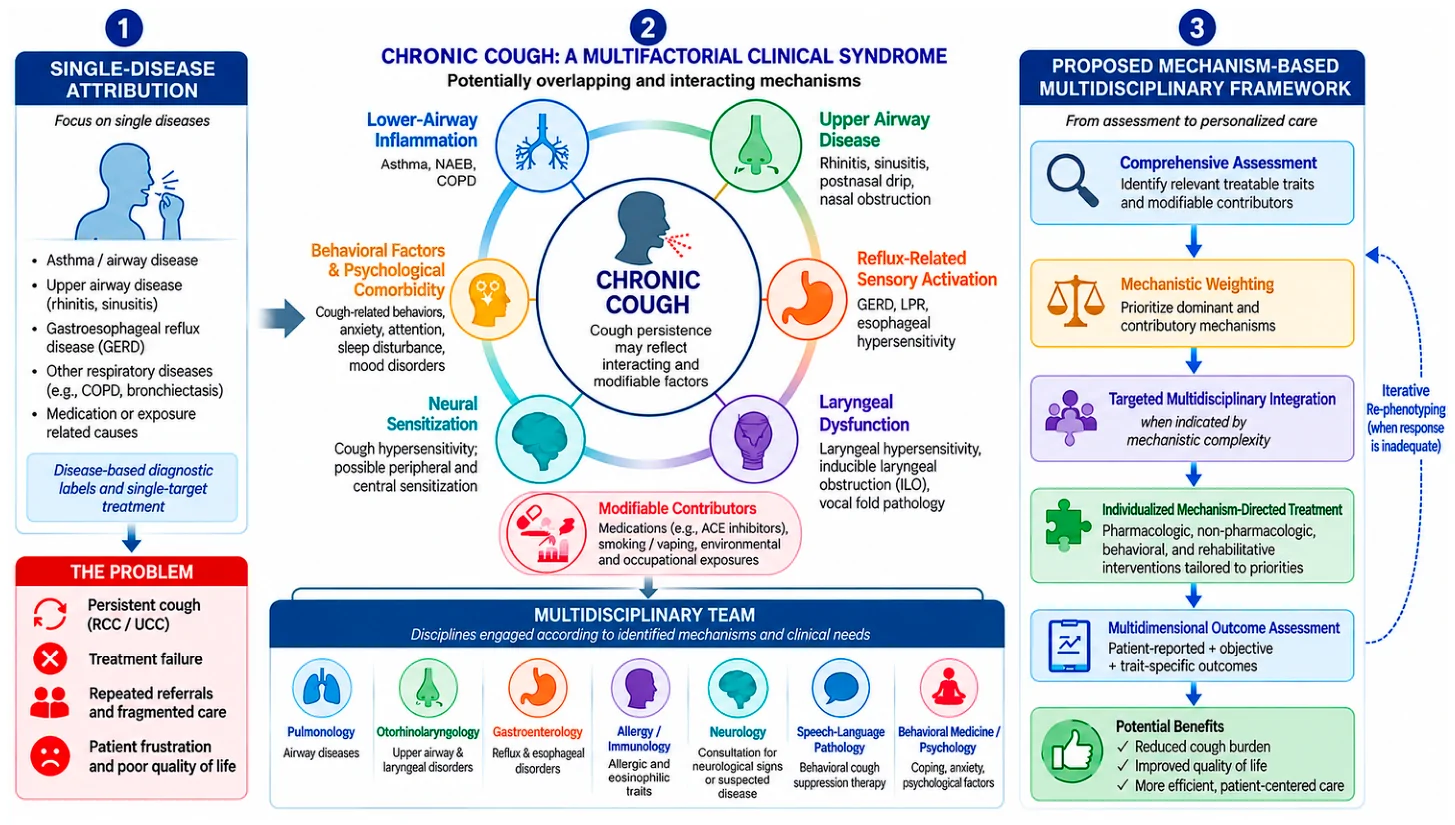
From Single-Disease Attribution to a Proposed Mechanism-Based Multidisciplinary Framework for Chronic Cough. The figure presents the authors’ conceptual synthesis of potentially overlapping cough contributors and a proposed mechanism-based care framework. Pragmatic mechanistic weighting is intended to guide targeted multidisciplinary involvement and individualized treatment, with inadequate response prompting reassessment. The complete framework is unvalidated; clinical and efficiency benefits require prospective evaluation (ACE, angiotensin-converting enzyme; COPD, chronic obstructive pulmonary disease; GERD, gastroesophageal reflux disease; ILO, inducible laryngeal obstruction; LPR, laryngopharyngeal reflux; NAEB, nonasthmatic eosinophilic bronchitis; RCC, refractory chronic cough; UCC, unexplained chronic cough).

Figure 1 summarizes this conceptual transition from disease-centered attribution toward mechanism-based assessment and individualized therapeutic prioritization.

## 6. Overlapping Mechanisms, Clinical Patterns, and Patient Modifiers

Existing studies illustrate that chronic cough can be subgrouped using different approaches. In a multicenter analysis of 255 adults, Kang et al. identified four clinical clusters using demographic characteristics and Cough Assessment Test (COAT) responses. The clusters differed in symptom burden and the distribution of associated diagnoses, demonstrating the potential of questionnaire-informed clinical phenotyping. However, the study did not establish whether cluster-based treatment selection improves outcomes, and external validation is required [42].

Long et al. reported a complementary physiological approach, identifying four response profiles among 234 patients who completed both capsaicin and allyl isothiocyanate challenges: hypersensitivity to both agents, either agent alone, or neither. The reported frequencies of cough triggers and laryngeal paraesthesia were similar across these groups, suggesting that these clinical features alone may not distinguish the physiological response profiles. These findings support heterogeneity in cough pathways but do not establish routinely applicable treatment-selection endotypes [43].

These overlapping features should not necessarily be interpreted as equivalent or mutually exclusive phenotypes. For clinical purposes, they may be more appropriately considered at different levels of classification, including mechanistic patterns, clinical-context patterns, and patient-specific modifiers. This distinction recognizes that some patterns primarily reflect biological mechanisms of cough persistence, whereas others describe the clinical context in which cough occurs or patient characteristics that may influence susceptibility, expression, or management. Therefore, the patterns proposed in this review are intended as non-exclusive clinical reasoning aids, not as a classification derived from a new cluster analysis or as validated molecular endotypes. Their selection reflects recurring clinical presentations, complementary assessment needs, and potential therapeutic relevance described in the literature [2,30,38,44,45,46,47,48,49]. Representative mechanistic patterns and clinical contexts relevant to chronic cough assessment are summarized in Table 3.

A commonly encountered multidimensional pattern involves the coexistence of asthma or eosinophilic airway disease, reflux-related mechanisms, and upper airway pathologies such as CRS or allergic rhinitis [2,17]. In such patients, treatment directed at only one mechanism may produce incomplete improvement because other active cough drivers remain unaddressed [2,10,33].

Another clinically important mechanistic pattern is characterized by prominent cough hypersensitivity, particularly among patients with RCC or UCC who remain symptomatic despite appropriate evaluation and treatment of identifiable conditions [4,6,10].

Management may include behavioral cough suppression therapy (BCST) and, when appropriate, pharmacological treatment targeting cough hypersensitivity [4,5,50,51].

A laryngeal hypersensitivity–dominant pattern may be suspected in patients with prominent throat sensations, globus, dysphonia, repetitive throat clearing, and cough triggered by phonation or vocal effort [37,38,52]. These features may coexist with inducible laryngeal obstruction (ILO), dysfunctional breathing, muscle tension dysphonia, or symptoms suggestive of laryngeal sensory involvement.

Paradoxical vocal fold motion (PVFM) describes paradoxical adduction of the vocal folds during breathing, whereas ILO encompasses symptomatic, transient narrowing at the glottic and/or supraglottic level. ILO may coexist with laryngeal or cough hypersensitivity but should not be equated with these sensory constructs [38,53]. Because obstruction may be absent between episodes, a normal resting laryngoscopy does not exclude ILO; diagnostic confirmation requires visualization of inappropriate narrowing during symptoms, with clinically appropriate provocation when necessary [53,54]. Isolated expiratory vocal fold adduction may also be observed in healthy individuals and should not, by itself, establish symptomatic ILO or explain cough persistence [54].

Importantly, symptoms may be prominent despite limited structural abnormalities on laryngeal examination, emphasizing the distinction between laryngeal dysfunction and laryngeal structural disease [2,12].

Recognition of this pattern is clinically important because targeted speech-language pathology and behavioral interventions may improve symptoms [51,55].

Other clinically relevant patterns are better defined by the context in which chronic cough develops or persists rather than by a single underlying mechanism. Respiratory infections are well-recognized triggers of prolonged cough, and in a subset of patients, cough persists beyond the resolution of the acute infectious illness [48].

Postviral cough, including cough following SARS-CoV-2 infection, may involve persistent sensory dysfunction and cough hypersensitivity after the acute infectious phase. Neuroinflammatory and neuroimmune interactions involving vagal sensory pathways have been proposed as contributing mechanisms [48].

Once active infection and other clinically relevant causes have been excluded, management may need to address persistent cough hypersensitivity rather than the initiating infection itself.

Older adults represent a clinically distinct population in which chronic cough must be interpreted in the context of swallowing function, frailty, neurological comorbidity, and airway protection. Age-related impairment of cough and swallowing reflexes may increase vulnerability to silent aspiration and aspiration pneumonia, particularly in frail individuals and those with neurological disorders [45,47].

In this population, chronic cough may arise from interacting mechanisms, including dysphagia and aspiration, GERD, chronic airway disease, polypharmacy, and age-related changes in cough reflex regulation. Impaired tongue propulsion and delayed laryngeal vestibule closure may further compromise swallowing efficiency and airway protection in frail older adults with dysphagia [56]. Importantly, chronic cough in older adults may reflect either excessive sensory activation or an airway-protective response to the dysphagia and aspiration. Therefore, distinguishing cough hypersensitivity from cough serving a protective function is essential before initiating cough-suppressive treatment, because suppression of an already compromised airway defense mechanism could potentially increase aspiration-related risk [45,47].

The evaluation of swallowing function and aspiration risk may be as important as traditional respiratory investigations in selected patients. When dysphagia, silent aspiration, or impaired airway protection is suspected, instrumental swallowing assessment using flexible endoscopic evaluation of swallowing (FEES) or videofluoroscopic swallowing study (VFSS) may provide clinically actionable information regarding swallowing physiology, pharyngeal residue, penetration, aspiration, and airway protection status [46,49].

Biological sex may act as a patient-specific modifier of cough susceptibility and sensory responsiveness. It should not be regarded as an independent mechanistic phenotype, a treatable trait, or a surrogate marker for cough hypersensitivity. Current evidence does not establish a basis for sex-specific treatment selection; management should be guided by clinically relevant mechanisms and treatable traits [15].

## 7. Current Diagnosis and Treatment: Challenges and Unmet Needs

Fragmented care may contribute to the complex and prolonged healthcare journey of patients with chronic cough. Community-based studies have demonstrated that patients may seek care from different healthcare professionals and specialties, while unmet needs remain regarding diagnostic evaluation, treatment effectiveness, and access to appropriate specialist care [57].

Laryngeal symptoms and functional abnormalities may be attributed solely to reflux or upper airway disease, whereas ILO, dysfunctional breathing, and laryngeal hypersensitivity remain unrecognized [37,38,52].

Conversely, nonspecific laryngoscopic abnormalities should not be automatically interpreted as evidence of a causal laryngeal or reflux-related disorder [2,12].

Diagnostic uncertainty may lead to extensive testing and repeated empirical treatment. Although comprehensive investigations are appropriate when clinically indicated, repeated or low-yield testing may increase healthcare utilization and costs without necessarily providing additional, clinically actionable information [9].

Similarly, empirical use of proton pump inhibitors, inhaled therapies, antibiotics, or other medications requires reassessment when response is inadequate. Continued treatment should depend on the indication, adequacy of the therapeutic trial, and evidence that the targeted condition or trait remains clinically relevant [2,23].

The healthcare burden of chronic cough is also substantial. Diagnostic investigations and specialist consultations are major contributors to cough-related healthcare costs [58], while patients with RCC or UCC have been reported to incur approximately threefold higher healthcare costs during the five years preceding specialist diagnosis than matched controls [59]. Population-based data similarly demonstrate greater healthcare resource utilization among adults with chronic cough, including more frequent healthcare visits and greater use of emergency, hospital, and selected specialist services [60].

In the prospective, single-center observational PROCOUGH study, guideline-based therapy was associated with improvements in objective cough frequency, cough severity, and cough-related QoL. Nevertheless, only 29% of the 100 patients completing follow-up reported adequate cough control [61]. Improvement in individual outcome measures therefore did not necessarily indicate adequate patient-perceived control.

Selected pharmacological and non-pharmacological interventions for RCC and UCC are summarized in Table 4.

Gabapentin is one of the best-studied neuromodulators and is recommended in international guidelines for selected patients with RCC [2,20,62].

Pregabalin has also demonstrated benefits in RCC when combined with speech-language pathology interventions, producing greater improvements in cough severity and cough-related QoL than speech-language pathology interventions alone [63]. Amitriptyline has shown potential benefits in a small randomized controlled study of patients with chronic cough attributed to postviral vagal neuropathy, although the limited evidence precludes broad generalization to RCC populations [64].

Gabapentin and pregabalin bind to α2δ subunits associated with voltage-gated calcium channels, as demonstrated in basic pharmacological studies; however, these findings do not establish the complete mechanism responsible for their clinical antitussive effects [65,66]. Amitriptyline has established interactions with monoamine transporters; however, whether these actions account for its reported benefit in selected patients with cough remains uncertain [64,67]. Clinical efficacy, pharmacological target engagement, and proof of a cough-specific mechanism should therefore be considered distinct levels of evidence.

Despite their potential benefits, neuromodulators are frequently associated with adverse effects, such as dizziness, fatigue, somnolence, and cognitive impairment. Therefore, their use should involve careful patient selection, individualized assessment of expected benefits and tolerability, and appropriate monitoring during the treatment [2,62].

Paroxetine, a selective serotonin reuptake inhibitor, has also been used in RCC, but supporting evidence is very limited. In a retrospective cohort, only two of seven patients receiving paroxetine after prior treatment trials showed a favorable response. These uncontrolled observations do not establish efficacy or support routine use in RCC/UCC [68].

Flupentixol/melitracen combines an antipsychotic with a tricyclic antidepressant; its cough-specific mechanism remains uncertain. In a single-center, double-blind trial, 102 patients with RCC unresponsive to prior neuromodulators were randomized, and 99 were analyzed. After two weeks of treatment and one week of follow-up, a ≥50% reduction in total cough symptom score was reported in 65.3% receiving active treatment versus 32.0% receiving placebo. The short duration, selected population, absence of objective cough monitoring, and greater somnolence limit generalization [69].

Low-dose slow-release morphine is another option for selected adults with RCC under specialist supervision. An early randomized placebo-controlled trial demonstrated improvement in cough-related QoL and daily cough scores without a significant change in citric-acid cough-challenge responses [70]. Its use requires an individualized assessment of benefits, constipation, sedation, and other opioid-related risks, with monitoring and reconsideration when meaningful benefits are absent [2,23].

More recently, a short-duration randomized crossover trial in 22 patients who had previously reported a clinical response to morphine found a 71.8% reduction in 24-h cough frequency versus placebo and improvement in experimentally evoked airway sensations, without a significant change in the citric-acid cough threshold [71]. These findings provide preliminary support for sensory modulation as an antitussive mechanism; however, the small responder-enriched population and brief treatment periods limit generalization to unselected patients with RCC.

P2X3 receptor antagonists target adenosine triphosphate (ATP)-mediated activation of vagal sensory neurons [21,50].

In the COUGH-1 and COUGH-2 trials, gefapixant 45 mg twice daily significantly reduced 24-h cough frequency compared with placebo in patients with RCC or UCC. However, the magnitude of the clinical benefit was modest, and taste-related adverse events, particularly dysgeusia, were frequently reported [72].

The clinical development of gefapixant has stimulated the investigation of more selective P2X3 receptor antagonists designed to preserve antitussive activity while reducing taste-related adverse effects associated with P2X2/3 receptor inhibition. Preclinical pharmacological studies of BLU-5937, which was subsequently developed as camlipixant, demonstrated potent and highly selective antagonism of homotrimeric P2X3 receptors, with substantially lower activity at P2X2/3 heterotrimeric receptors. This selectivity provides a mechanistic rationale for reducing taste disturbance while inhibiting ATP-mediated sensory signaling relevant to cough hypersensitivity [73].

Consistent with this pharmacological rationale, camlipixant has demonstrated clinical activity in the phase 2b SOOTHE trial. In adults with RCC, treatment with camlipixant 50 mg and 200 mg twice daily produced statistically significant placebo-adjusted reductions in the 24-h objective cough frequency. Patient-reported measures of cough severity and cough-related QoL also showed improvement, whereas taste-related adverse events occurred in a relatively small proportion of patients [74].

According to the sponsor’s phase III results announcement, CALM-1 met its primary endpoint with camlipixant 50 mg twice daily, showing a significant reduction in 24-h cough frequency versus placebo at week 12, whereas CALM-2 did not reach statistical significance with the same dose at week 24. The 25 mg twice-daily dose did not reach statistical significance in either trial, and the key secondary endpoints did not meet the target thresholds. Based on the aggregate findings, the sponsor discontinued further development of camlipixant for RCC [75].

Pharmacological selectivity alone does not establish clinical effectiveness. Dose–response and comparative evidence suggest that receptor selectivity, dosing, and pharmacological properties may influence the balance between antitussive efficacy and taste-related adverse events [76,77].

BCST and speech-language pathology interventions are important non-pharmacological treatment options for selected patients with RCC and UCC, particularly those with laryngeal hypersensitivity, urge-to-cough sensations, phonation-triggered cough, dysfunctional breathing, or difficulty in voluntarily suppressing cough [21,38,78].

Structured interventions typically incorporate cough suppression strategies, laryngeal desensitization, respiratory retraining, vocal hygiene education, and behavioral modifications [22,38].

Evidence syntheses have demonstrated improvements in cough-related outcomes following structured speech-language pathology interventions, including reductions in cough frequency and improvements in cough-related QoL. Combination with neuromodulatory therapy may provide additional benefits to selected patients with refractory symptoms [55,63,78,79]. Follow-up studies have suggested that therapeutic benefits may persist beyond treatment completion in some patients [80].

In a multicenter randomized trial, four weekly sessions of physiotherapy and speech and language therapy intervention (PSALTI) improved cough-related QoL and reduced objective cough frequency by 41% relative to control at four weeks. Improvements within the PSALTI group persisted at three months, although sustained between-group superiority was not demonstrated [51,55].

These diagnostic and therapeutic limitations inform the mechanism-based multidisciplinary framework proposed in Section 8.

## 8. A Mechanism-Based Multidisciplinary Framework for Chronic Cough Care

### 8.1. Scope, Conceptual Basis, and Core Principles

The proposed framework is intended to complement guideline-based care in adults whose persistent cough is difficult to manage because coexisting traits, discordant findings, or inadequate treatment responses complicate therapeutic prioritization [23].

The framework proposed in this review is an evidence-informed conceptual clinical model rather than a validated diagnostic algorithm or a standard of care. Established disease evaluation, assessment of treatable traits, selected laryngeal and swallowing assessments, and pharmacological and behavioral interventions are supported to varying degrees by the literature discussed in this review. Their integration into the specific nine-step pathway and proposed mechanistic-weighting categories represents the authors’ conceptual synthesis and requires prospective validation.

### 8.2. A Flexible Multidisciplinary Team

The composition of the multidisciplinary team should be flexible rather than fixed and should reflect the mechanisms and treatable traits identified in each patient. The goal is not to require every patient to be assessed by every discipline but to involve the expertise needed to resolve specific diagnostic and therapeutic uncertainties.

A core chronic cough team may include pulmonology, otorhinolaryngology, and speech-language pathology, because these disciplines collectively address airway disease, upper airway and laryngeal function, cough hypersensitivity, breathing patterns, and behavioral cough control. Gastroenterology, allergy, behavioral medicine or psychology, geriatrics, sleep medicine, physiotherapy, swallowing specialists, occupational medicine, clinical pharmacology, and pharmacy expertise may be incorporated according to the patient’s phenotype and identified traits [12,81]. Within this framework, neurology consultation is reserved for patients with relevant neurological signs or a suspected coexisting neurological disorder.

The shared information should include medication and exposure histories, therapeutic decisions, objective measurements, and patient-centered goals [23,82].

Speech-language pathology contributes to both treatment and functional assessment. Evaluation of cough triggers, voluntary cough control, phonation-related symptoms, breathing patterns, laryngeal behaviors, and urge-to-cough characteristics can help characterize functional symptom patterns and identify targets for behavioral treatment [22,38,51,79]. It may therefore contribute early to assessment and treatment planning in appropriately selected patients.

### 8.3. A Proposed Mechanism-Based Diagnostic and Management Pathway

The pathway is designed as an iterative rather than a strictly linear process: clinical findings generate mechanistic hypotheses, targeted interventions test these hypotheses, and treatment responses subsequently refine the patient’s mechanistic phenotype.

#### 8.3.1. Step 1: Confirm Chronic Cough, Identify Red Flags, and Perform Comprehensive Medication and Exposure Reviews

The pathway begins with confirmation that the cough fulfills the definition of chronic cough and systematic screening for features suggesting serious or potentially reversible underlying diseases. Relevant warning features may include hemoptysis, unexplained weight loss, progressive dyspnea, recurrent pneumonia, persistent chest pain, abnormal chest imaging, or other findings suggesting malignancy, significant respiratory disease, infection, aspiration, or other conditions that require prompt targeted investigation [2,10]. Patients with such features should undergo appropriate diagnostic evaluation before proceeding through a mechanism-based chronic cough pathway.

A comprehensive medication review should be part of this initial assessment and should extend beyond the conventional evaluation of angiotensin-converting enzyme (ACE) inhibitor use. ACE inhibitors remain the best-established pharmacological cause of chronic cough and may enhance cough-reflex sensitivity through mechanisms involving impaired degradation of bradykinin and substance P. Importantly, cough may develop shortly after treatment initiation or only after weeks or months of exposure; therefore, the absence of a clear temporal relationship between the initiation of therapy and cough onset does not exclude a medication-related contribution [6,83].

Other medications should be considered when clinically relevant, although the strength of evidence is substantially lower than that for ACE inhibitors. Dipeptidyl peptidase-4 (DPP-4) inhibition, particularly with sitagliptin, has been associated with cough and respiratory symptoms in some studies, but this relationship has not been consistently reproduced and may reflect host–drug interactions in susceptible individuals. Calcium channel blockers may contribute indirectly by aggravating gastroesophageal reflux, whereas isolated observations suggest that other pharmacological agents may alter cough reflex sensitivity through different mechanisms. Importantly, the medication history should include all routes of administration, including oral, inhaled, topical, and ophthalmic therapies. A case report of increased cough-reflex sensitivity associated with ophthalmic latanoprost illustrates the importance of including ophthalmic therapies in the medication history [83].

A 2025 analysis of the FDA Adverse Event Reporting System (FAERS) identified disproportionate reporting of cough across multiple drug classes, including antineoplastic and immunomodulating agents, respiratory system medications, and cardiovascular drugs. The reported outcome was cough, not specifically chronic cough. These signals are hypothesis-generating; reporting biases, confounding by indication, concomitant medications, underlying diseases, and differences in drug utilization limit causal interpretation in an individual patient [84].

Taken together, mechanistic evidence and contemporary pharmacovigilance data suggest that medication-related cough should not uniformly be interpreted as a simple direct adverse drug reaction. Depending on the drug, host susceptibility, underlying disease, and cough phenotype, pharmacological exposure may function as a causal factor, trigger, amplifier, or contributor to cough [83,84]. Except for well-established associations, such as ACE inhibitor-related cough, causal attribution should remain cautious and should consider biological plausibility, temporal relationships, alternative explanations, and response to clinically appropriate medication modifications.

A similarly comprehensive assessment should address smoking, vaping, and other relevant environmental and occupational exposures. Current and previous tobacco smoking, secondhand smoke exposure, electronic cigarette use, exposure to combustion products, traffic-related air pollution, workplace dust, fumes, vapors, chemicals, allergens, and other inhaled irritants should be documented. Such exposures may contribute to chronic cough through several pathways, including airway inflammation, allergic or occupational airway disease, direct irritant effects, and sensory-neural activation [6,85].

The distinction between an exposure that causes disease and an exposure that triggers cough in a sensitized system is particularly important within a mechanism-based framework [6]. Environmental exposures may act as causal factors in some patients but as triggers or amplifiers of an established hypersensitive cough network in others.

Occupational history should extend beyond simply recording a patient’s occupation. The nature, intensity, and duration of relevant exposures should be considered, along with the temporal relationship between exposure and the symptoms. Improvement during weekends, holidays, or periods away from work, or deterioration after returning to a particular occupational environment, may provide clinically useful information and should prompt further investigation of occupational airway disease when appropriate. Suspected occupational airway disease may require objective investigations and specialist occupational assessment [85].

Electronic cigarette use should be specifically documented as part of a contemporary exposure assessment. Although the long-term relationship between vaping and specific chronic cough phenotypes remains incompletely characterized, contemporary evidence associates e-cigarette use with adverse respiratory outcomes. The certainty of evidence for several reported associations remains low; therefore, vaping should be considered a potentially relevant inhalational exposure rather than being assumed to represent either a proven cause of cough hypersensitivity or a benign exposure [86].

#### 8.3.2. Step 2: Establish Baseline Cough Burden and Characterize the Clinical Pattern

Following the initial safety, medication, and exposure assessments, the clinical burden and pattern of cough should be systematically characterized. The evaluation should document cough duration, frequency, and severity; diurnal and nocturnal patterns; characteristic triggers; urge-to-cough sensations; sputum production; associated respiratory symptoms; upper airway or laryngeal complaints; voice changes; swallowing symptoms; functional impairment; sleep disturbance; and the physical, psychological, and social consequences of cough [1,2,23].

The history should also include previous investigations, therapeutic trials, adherence, and the degree and duration of treatment response. Rather than merely recording whether previous treatment was successful or unsuccessful, clinicians should determine which component of the cough improved, to what extent, and for how long, because partial responses may provide mechanistic information regarding the contributory traits of the cough.

Standardized patient-reported measures, such as the Leicester Cough Questionnaire (LCQ), along with cough severity scales and objective cough monitoring, when available, can provide a reproducible baseline against which subsequent treatment responses can be assessed [87,88,89].

Questionnaires can also support descriptive phenotyping rather than solely quantifying symptom burden. The Cough Hypersensitivity Questionnaire (CHQ), developed through patient interviews and multidisciplinary review and evaluated in 535 patients, characterizes six cough-related sensations and 16 triggers. The initial CHQ study supported validity but identified repeatability, responsiveness, and clinical utility as priorities for further evaluation [90]. The 18-item Cough Hypersensitivity Assessment Test (CHAT) covers environmental triggers, daily life triggers, and tussive symptoms, with evidence supporting reliability, construct validity, and responsiveness [91]. The 14-item Newcastle Laryngeal Hypersensitivity Questionnaire assesses laryngeal sensory symptoms and may complement functional assessment when laryngeal involvement is suspected [92]. These instruments address different domains from cough-related QoL and should be selected according to the clinical or research question.

Questionnaire development and evaluation should incorporate patient-derived content, cognitive debriefing, appropriate item reduction, and assessments of validity, reliability, and responsiveness. Validation in the intended language and population is important before scores or proposed thresholds are transferred across settings. Questionnaire profiles may help identify symptom patterns, guide further assessment, and monitor changes; however, psychometric validity should not be equated with the confirmation of a specific neural lesion, molecular endotype, or dominant cough mechanism [90,91,92].

#### 8.3.3. Step 3: Identify Established Diseases, Treatable Traits, and Modifiable Contributors

The assessment should retain a guideline-based respiratory foundation, including chest radiography and diagnostic spirometry, with blood eosinophil assessment and fractional exhaled nitric oxide (FeNO), where available and clinically relevant. Additional imaging and specialized investigations should be selected according to clinical findings rather than being applied indiscriminately [2,23].

Potential traits and contributors may include eosinophilic airway inflammation, asthma or other bronchial diseases, CRS or allergic upper airway diseases, reflux-related sensory activation, laryngeal dysfunction, cough hypersensitivity, dysfunctional breathing, obesity, sleep-disordered breathing, aspiration or swallowing dysfunction in selected populations, psychological comorbidity and behavioral factors that may influence symptom burden or cough control, medication-related cough or pharmacological amplification of other active mechanisms, and clinically relevant environmental or occupational exposures [6,28,83,85].

#### 8.3.4. Step 4: Characterize Cough Hypersensitivity and Laryngeal Involvement

Assess cough hypersensitivity features, including allotussia, hypertussia, an exaggerated urge to cough, and difficulty suppressing cough voluntarily [4,10,21].

Associated laryngeal features, including throat irritation or tickling, globus sensation, dysphonia, frequent throat clearing, phonation-induced cough, abnormal breathing patterns, and symptoms suggestive of ILO, should also be sought [37,38,52]. When clinically indicated, laryngeal examination can assess structural abnormalities and vocal-fold function; laryngoscopy with appropriate provocation can confirm suspected ILO. These findings do not, by themselves, establish cough hypersensitivity or localize an underlying sensory-neural abnormality [23,53].

#### 8.3.5. Step 5: Weight the Clinical Relevance of Identified Traits and Contributors

The identification of a disease, treatable trait, medication exposure, environmental factor, or diagnostic abnormality should not be automatically equated with causal attribution. Each finding should be interpreted according to its biological plausibility, temporal relationship with cough, objective support, consistency with the overall clinical phenotype, previous therapeutic responses, and interactions with other active mechanisms [23].

Within the proposed framework, identified traits and contributors may be pragmatically considered dominant, contributory, incidental, or currently inactive according to their probable relevance to cough persistence. A dominant trait is likely to account for a substantial component of the current cough burden; a contributory trait may amplify or perpetuate cough without independently explaining the entire syndrome; an incidental finding is judged unlikely to contribute meaningfully to the current cough after adequate assessment; and a currently inactive trait represents a previously relevant mechanism that is adequately controlled or no longer appears to contribute substantially to the current symptoms.

These categories are proposed as a pragmatic clinical reasoning framework rather than validated diagnostic classifications and require prospective evaluation.

In practical application, weighting should be based on converging evidence rather than isolated abnormalities. The supporting and conflicting clinical, objective or functional, temporal, and treatment-response findings, the degree of uncertainty, and the reasons for revising a weight should be documented. The framework does not provide validated cut-offs for the four categories; assignments should therefore remain provisional and be reconsidered during follow-up. Inter-rater agreement across clinicians and specialties, reproducibility over time, and the clinical benefit of structured weighting require prospective evaluation.

Available tests support different parts of this assessment. Sputum eosinophils, blood eosinophils, and FeNO may support an inflammatory trait but do not establish its relative contribution to cough [2,36]. Functional laryngoscopy may identify inducible laryngeal obstruction, while appropriately selected reflux investigations can characterize reflux burden; neither an isolated laryngeal finding nor evidence of reflux automatically establishes cough causality [2,23,33]. Questionnaires and objective cough monitoring characterize symptoms and change over time rather than independently assign mechanistic weight. A finding that has not been adequately evaluated should remain unclassified or uncertain rather than automatically being labelled incidental or inactive.

For illustration, in a hypothetical patient with eosinophilic airway inflammation and phonation-triggered cough, improvement in inflammatory findings without meaningful cough improvement would justify reconsidering the initial weighting and reassessing the coexisting laryngeal or sensory features. Conversely, the persistence of both inflammation and cough would first require an assessment of treatment adequacy and adherence. Such reasoning should remain provisional and account for competing mechanisms and nonspecific treatment effects; it does not constitute the validation of the proposed categories.

#### 8.3.6. Step 6: Determine the Need for Targeted Multidisciplinary Integration

Patients with a single dominant and readily treatable mechanism may continue to receive conventional, specialty-based care. Therefore, multidisciplinary assessment should not be regarded as mandatory for all patients with chronic cough [23].

Multidisciplinary integration should be considered early when indicators of clinical complexity are present, rather than being reserved exclusively for patients who have undergone multiple unsuccessful sequential treatment trials.

Targeted multidisciplinary integration may be considered when multiple potentially active traits coexist, findings from different organ systems are discordant, symptoms appear disproportionate to objective abnormalities, treatment responses are incomplete or contradictory, or prominent laryngeal, swallowing, neural, behavioral, pharmacological, environmental, or occupational mechanisms complicate causal interpretation [23,81,82].

The disciplines involved should be selected according to the patient’s mechanistic profile rather than through routine referral to a fixed panel of specialists [23,81].

#### 8.3.7. Step 7: Select and Prioritize Mechanism-Directed Interventions

Treatment should target the mechanisms and modifiable contributors that are considered to be the most clinically relevant. Depending on the patient’s phenotype, interventions may include anti-inflammatory treatment for eosinophilic airway disease, management of upper airway pathology, appropriately selected reflux-directed therapy, BCST, speech-language pathology interventions, neuromodulatory treatment, P2X3-directed therapies when appropriate and available, management of dysfunctional breathing, or swallowing and aspiration interventions in selected patients [2,20,23,51,62,63,72].

When medication exposure is suspected to contribute to cough, withdrawal, substitution, dose modification, or other adjustments of the suspected medication may be considered when clinically appropriate and coordinated with the relevant prescribing clinician. Such modifications should be regarded as mechanism-directed therapeutic trials rather than proof of causality [83,84,85].

Similarly, when environmental or occupational exposure is considered clinically relevant, appropriate exposure reduction or avoidance should be incorporated into the management plan when feasible [85].

In the presence of several active mechanisms, therapeutic priorities should reflect their estimated relative contributions, potential reversibility, strength of supporting evidence, safety, and feasibility rather than attempting to treat every identified abnormality simultaneously. Treatment selection should also incorporate patient preferences, comorbidities, the anticipated benefit–burden balance, the accessibility of the proposed intervention, and individual therapeutic goals [23].

#### 8.3.8. Step 8: Reassess Using Patient-Centered, Objective, and Trait-Specific Outcomes

Treatment response should be assessed using a combination of patient-reported outcomes, cough severity, cough-related QoL, objective cough frequency when available, functional outcomes, and response to specific traits or contributors targeted by therapy [87,88,93].

Additional outcomes may include medication burden, healthcare utilization, patient satisfaction, and achievement of individualized treatment goals. Clinically meaningful improvement may occur even when cough does not completely resolve.

Importantly, objective cough frequency and patient-reported cough burden capture related but distinct dimensions of chronic cough and should therefore be regarded as complementary rather than interchangeable [88,89,93].

Digital and artificial intelligence (AI)–assisted cough monitoring should support clinical interpretation using systems validated for their intended setting. Differences in cough-event definitions, reference standards, and real-world performance limit comparisons across systems and require further standardization [94].

Response of the targeted trait should be assessed separately from cough response. Improvement in the trait without meaningful cough improvement should prompt reconsideration of its contribution to the current cough and assessment of competing contributors; it should not automatically lead to classification as incidental. If neither the trait nor cough improves, treatment adequacy and adherence should be reviewed before concluding that the initial mechanistic attribution was incorrect [23].

Medication withdrawal or substitution and environmental or occupational exposure modification may function as therapeutic tests when clinically appropriate. Improvement following modification strengthens—but does not necessarily prove—the proposed relationship, whereas persistent cough should prompt reconsideration of causal attribution and investigation of competing mechanisms [84,85].

#### 8.3.9. Step 9: Re-Phenotype When Response Is Inadequate

An inadequate or incomplete response should not automatically lead to the escalation of the same therapy or an unrestricted search for increasingly rare diagnoses. Instead, it should trigger a structured re-evaluation of the mechanistic models.

Reassessment should address treatment adherence and adequacy, persistence of the targeted trait, incorrect weighting of previously identified mechanisms, competing or newly emerging traits, medication exposure, smoking or vaping, environmental and occupational exposures, laryngeal or sensory-neural mechanisms, swallowing dysfunction where relevant, and previously unrecognized contributors [23].

**Figure 2:**
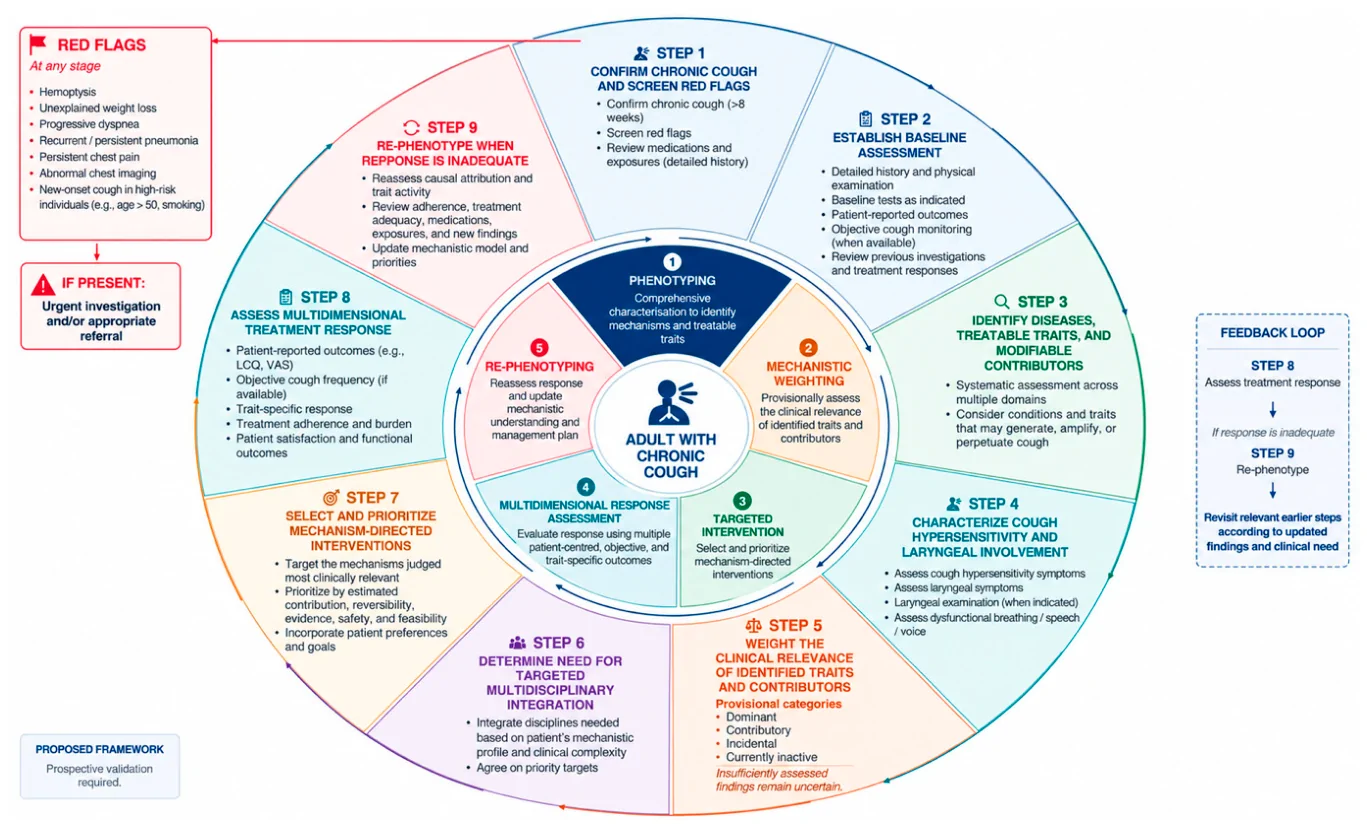
Proposed Integrated and Iterative Mechanism-Based Model for Multidisciplinary Chronic Cough Care. The outer ring presents the nine-step adult pathway; the inner cycle links phenotyping, provisional mechanistic weighting, targeted intervention, response assessment, and re-phenotyping. Red flags prompt urgent investigation or appropriate referral at any stage. Multidisciplinary involvement is selected according to clinical need. The pathway and weighting categories require prospective validation (LCQ, Leicester Cough Questionnaire; VAS, visual analog scale).

The nine-step pathway and its iterative reasoning cycle are summarized in Figure 2.

### 8.4. Special Considerations in Pediatric Chronic Cough

Although the mechanism-based multidisciplinary framework proposed in this review is primarily intended for adults, chronic cough in children deserves specific consideration because adult diagnostic concepts and therapeutic strategies cannot be directly extrapolated to the pediatric population. Chronic cough in children is generally defined as a daily cough persisting for more than 4 weeks, rather than the 8-week threshold commonly used in adults, and pediatric-specific diagnostic algorithms are recommended for its diagnosis [2,95]. Differences in airway anatomy and physiology, maturation of cough and swallowing reflexes, patterns of underlying disease, and the clinical significance of cough itself require a distinct diagnostic hierarchy.

In children, the primary objective remains the identification of an underlying cause rather than early attribution of persistent cough to cough hypersensitivity. The distinction between wet and dry cough is particularly informative, and chronic wet cough should prompt consideration of protracted bacterial bronchitis (PBB), bronchiectasis, other suppurative airway diseases, aspiration, airway abnormalities and persistent infection [2,96]. Foreign body aspiration, congenital airway abnormalities, swallowing dysfunction, and other age-specific disorders should also be considered according to the clinical context. PBB is especially relevant in younger children and represents an important example of a treatable pediatric cough condition in which appropriate etiological treatment may resolve cough [96].

Children require etiological assessment rather than routine application of the adult CHS construct. Cough may be protective in airway infection, retained secretions, aspiration, or structural airway disease. Adult neuromodulator and sensory-neural treatment strategies should not be extrapolated to pediatric chronic cough without age-specific evidence [2,97].

Nevertheless, the broader principle of mechanism-based multidisciplinary care remains relevant for selected children, particularly those with complex aerodigestive disorders. Pediatric chronic cough may involve interacting lower-airway, upper airway, gastrointestinal, swallowing, nutritional, and structural mechanisms that cannot always be adequately evaluated within a single specialty. Pediatric aerodigestive models that integrate pulmonology, otorhinolaryngology, gastroenterology, speech-language pathology, and other disciplines provide an established framework for the coordinated assessment of children with complex breathing, swallowing, and feeding disorders [98,99,100].

The principles of coordinated assessment and response-guided reassessment may inform pediatric-specific diagnostic pathways, but the adult nine-step model and weighting categories require separate evaluation before pediatric application.

The potential advantages, practical constraints, and requirements for the validation of the proposed framework are considered in the following section.

## 9. Potential Advantages and Real-World Limitations of the Proposed Framework

The proposed framework may support more explicit therapeutic prioritization by linking provisional mechanistic weighting to coordinated treatment decisions and planned reassessment.

In a retrospective quality-improvement evaluation, a multidisciplinary collaborative care redesign was associated with fewer specialist referrals and a shorter time to complete evaluation than the existing care process [101]. An etiology-oriented multidisciplinary cough service also reported short-term improvement in RCC outcomes; however, the observational design and brief assessment period do not establish superiority over conventional care [82].

The principal limitation of the proposed framework is that the integrated model itself has not been prospectively validated. Although many of its individual components are supported by guidelines, clinical trials, observational studies, and contemporary treatable-trait and cough-hypersensitivity concepts, there is currently no evidence that applying the complete nine-step pathway improves clinical outcomes, diagnostic efficiency, healthcare utilization, or cost-effectiveness compared with established guideline-based care.

Provisional mechanistic weighting depends on clinical synthesis and may therefore be vulnerable to inter-clinician variability. No validated thresholds currently define the proposed categories, and the absence of sufficiently specific biomarkers for several proposed contributors, including cough hypersensitivity and suspected laryngeal sensory involvement, limits objective attribution.

The implementation may also be resource-intensive. Access to dedicated cough services, speech-language pathology, functional laryngeal assessment, swallowing evaluation, specialized reflux testing, objective cough monitoring, and relevant multidisciplinary expertise varies substantially across healthcare systems. A framework requiring extensive specialist involvement could inadvertently increase costs, waiting times, or healthcare inequity if applied indiscriminately. Therefore, the model is intended to support targeted rather than universal multidisciplinary integration. Practical implementation can use a designated coordinating clinician, shared documentation of mechanistic hypotheses and treatment priorities, and an agreed follow-up plan to reduce duplication across referrals. Before an intervention is classified as ineffective, adherence, access, tolerability, and the patient’s ability to implement the treatment should be reviewed.

There is also a potential risk that a broad mechanism-based assessment could generate additional testing or identify multiple abnormalities of uncertain relevance. Therefore, the framework depends on disciplined causal interpretation: the identification of a trait, biomarker abnormality, exposure, or functional finding should not automatically trigger treatment or specialist referral. Its clinical value will depend on whether mechanistic weighting can reduce, rather than simply reorganize, diagnostic complexity.

Finally, repeated assessments, multimodal interventions, and longitudinal monitoring may increase patient burden and require sustained engagement. Therefore, patient preferences, treatment burden, accessibility, comorbidities, and therapeutic goals should remain integral to prioritization decisions.

Prospective studies should evaluate the framework’s reproducibility, feasibility, clinical effectiveness, cost-effectiveness, and added value over existing care pathways. These priorities are discussed in Section 10.

## 10. Future Directions: Toward Precision Medicine in Chronic Cough

Future research should establish whether mechanistic characterization improves treatment selection and patient outcomes. Table 5 summarizes the key research gaps, their clinical relevance, and potential study designs to address them.

Future research should move beyond cataloguing treatable traits to define mechanistic endotypes. Such endotypes should be supported by reproducible evidence of a specific biological mechanism. Laryngeal symptoms, behavioral characteristics, and environmental exposures may inform clinical phenotyping and mechanistic hypotheses but do not, by themselves, establish an endotype.

Emerging evidence also raises the possibility that cough hypersensitivity may coexist with broader sensory hypersensitivity phenotypes in selected patients. In a prospective cohort study, sensitive skin syndrome was more prevalent among patients with RCC or UCC than among other patients with chronic cough and was associated with heightened cough sensitivity and differences in response to neuromodulatory treatment. These observations suggest that shared sensory processing characteristics may contribute to future biomarker- or phenotype-guided stratification. However, the observational treatment comparison did not establish the sensitive skin syndrome as a validated predictor of neuromodulator response [102].

One of the major unmet needs in chronic cough management is the absence of validated biomarkers capable of objectively identifying cough hypersensitivity, defining clinically relevant endotypes and predicting therapeutic responses [10,103,104].

Among the currently available biomarkers, those reflecting type 2 airway inflammation are the most clinically applicable. Induced sputum eosinophilia remains an important reference measure for identifying eosinophilic airway inflammation, a treatable trait frequently encountered in cough-variant asthma and non-asthmatic eosinophilic bronchitis [2,36]. However, sputum induction is labor-intensive and not widely available in routine clinical practice.

Consequently, surrogate biomarkers such as blood eosinophil count (BEC) and fractional exhaled nitric oxide (FeNO) have attracted increasing attention. In a prospective study involving 658 patients with chronic cough, BEC demonstrated moderate diagnostic accuracy for identifying sputum eosinophilia, whereas the combination of BEC and FeNO achieved greater diagnostic accuracy for identifying sputum eosinophilia, supporting a multimodal approach rather than a single-biomarker approach [36].

Inflammatory biomarkers identify inflammatory traits but do not establish cough hypersensitivity or its underlying neural mechanisms. Preclinical studies implicate peripheral and central sensory pathways in experimental cough sensitization [105,106]. Despite these mechanistic advances, no validated neural biomarker is currently available for routine clinical identification or quantification of cough hypersensitivity [103,107].

Emerging genetic evidence may provide an additional dimension to mechanistic phenotyping in patients with RCC. Biallelic repeat expansions in the replication factor C subunit 1 (RFC1) gene, classically associated with cerebellar ataxia with neuropathy and vestibular areflexia syndrome (CANVAS), have been identified in a subset of patients with RCC. In one cohort of 51 patients with RCC, four (8%) had biallelic RFC1 repeat expansions and demonstrated cough reflex hypersensitivity. Cough severity and cough-related quality of life did not significantly distinguish these patients from those without biallelic expansions. These findings suggest that genetically defined sensory-neural phenotypes may account for a subset of otherwise unexplained RCC and warrant further investigation within precision cough frameworks [108].

Emerging molecular approaches, including transcriptomic and other multi-omics strategies, may contribute to the identification of biologically distinct cough endotypes and predictors of therapeutic response. Transcriptomic analysis of cultured airway smooth muscle cells from a small chronic cough cohort identified altered responses to the viral mimic poly(I:C), whereas baseline differences in individual gene expression between patients and controls were not significant after multiple-testing correction [109]. More broadly, the integration of genomic, transcriptomic, proteomic, and metabolomic approaches with physiological measurements, clinical traits, and digital biomarkers may facilitate biomarker discovery and more refined patient stratification. However, these approaches remain exploratory for chronic cough, and their reproducibility, disease specificity, predictive value, and clinical utility require prospective validation before being incorporated into precision cough pathways [107].

Objective cough monitoring technologies provide a complementary approach for cough assessment. Systems such as the Leicester Cough Monitor and VitaloJAK have established the feasibility of objective cough frequency assessment and demonstrated that objective cough counts and patient-reported outcomes capture the related but distinct dimensions of chronic cough burden [88,89,110].

Advances in wearable devices, smartphones, and automated cough detection have expanded the potential for longitudinal monitoring. Such monitoring may characterize circadian variation, cough clustering, and treatment-related changes; interpretation of possible triggers or environmental associations requires relevant contextual data [89,110]. A smartphone-based longitudinal study demonstrated high adherence and substantial day-to-day variability in cough frequency, with variable relationships between objective measurements and patient-reported severity [111].

These developments have contributed to the concept of digital phenotyping, in which longitudinal patterns of cough behavior are interpreted in conjunction with conventional variables. The related concept of cough-omics extends this approach by integrating cough frequency, acoustic characteristics, temporal patterns, contextual information, and clinical data obtained from the natural environment [110]. Such multidimensional datasets may reveal phenotypic patterns that cannot be identified through conventional episodic assessments alone.

AI and machine learning approaches may further enhance the analyses of these complex datasets. Automated acoustic systems can support cough detection and quantification [89,112,113]. Separate machine-learning approaches using administrative or clinical data have been investigated for identifying chronic cough or predicting its persistence [114,115]. Preliminary conference data suggest that AI-assisted systems may also facilitate prolonged objective monitoring beyond conventional recording periods, potentially providing a more representative picture of the real-world cough burden [116].

However, the immediate clinical value of these technologies is likely to lie primarily in measurement and decision support rather than autonomous diagnosis. Claims that acoustic characteristics alone can reliably identify cough etiology or determine treatment should be interpreted cautiously until they are externally validated in diverse populations, devices, languages, and clinical settings [94,112,117].

Future studies should establish minimum technical standards, appropriate recording duration, clinically meaningful changes in objective cough frequency, and reproducibility across platforms. They should also test whether digital monitoring improves clinical decisions and patient-important outcomes. AI models require external and prospective validation, alongside attention to interoperability, privacy, and data governance [89,93,94,112,117].

Future therapeutic development should distinguish biological target engagement from clinically meaningful antitussive benefit [50,72,73,74,75,76,77].

Beyond P2X3 signaling, transient receptor potential channels, particularly TRPV1 and TRPA1, are important targets in cough research because of their roles in peripheral sensory activation and in sensitization. Experimental and clinical studies have demonstrated altered activity of these pathways in chronic cough, although therapeutic translation has been less successful than initially anticipated [10,24,50]. Nevertheless, these findings continue to provide important insights into airway sensory neurobiology and may inform future combination or phenotype-directed strategies.

Voltage-gated sodium channels, particularly NaV1.7 and NaV1.8, have attracted interest because of their roles in the initiation and propagation of action potentials in vagal sensory neurons. In guinea pig models, NaV1.7 inhibition and combined NaV1.7/NaV1.8 inhibition suppressed experimentally evoked cough [118,119,120]. Systemic administration of Compound 801 also produced hypotension and respiratory depression in guinea pigs, highlighting the importance of delivery and safety in further translation [120].

Central mechanisms are potential therapeutic targets. In conscious guinea pigs, intracerebroventricular prostaglandin E2 (PGE2) enhanced citric acid-evoked cough, with pharmacological experiments implicating EP3 receptors and NaV1.8 channels [105]. In a juvenile guinea pig model, activation of substance P-expressing neurons in the hypothalamic paraventricular nucleus was associated with persistent cough hypersensitivity, with tracing findings suggesting a hypothalamic–airway pathway. This preclinical observation warrants further investigation but does not establish a validated human biomarker or therapeutic target [121].

Advances in airway neurobiology have highlighted the heterogeneity among vagal sensory neurons, including the jugular and nodose pathways, which have distinct molecular and functional characteristics. More selective modulation of pathological sensory pathways may ultimately offer the possibility of reducing excessive cough while preserving physiologically important airway-protective reflexes [122].

Inflammatory precision therapies require cough-specific evaluation. In a prospective uncontrolled study, dupilumab was associated with improved patient-reported cough and cough-related quality of life in patients with severe eosinophilic asthma and/or chronic rhinosinusitis with nasal polyps [123]. In the randomized, placebo-controlled MUCOSA trial, mepolizumab did not significantly improve objective 24-h cough frequency in patients with RCC and persistent eosinophilic airways disease, despite reductions in blood and sputum eosinophils [124]. The different populations, targets, and study designs preclude a direct comparison between these biologics; inflammatory biomarker suppression should not be assumed to confer antitussive benefit.

Future trials should assess whether biological, sensory, laryngeal, behavioral, or digital phenotypes identify patients more likely to benefit from sensory-neural therapies [77,104,125]. Candidate response predictors should be evaluated through prespecified treatment-by-phenotype interaction analyses and independent validation. Future studies should incorporate predefined sex-stratified analyses and investigate whether biological sex, menopausal status, hormonal factors, sensory-neural phenotypes, or their interactions predict cough severity, persistence, and therapeutic response.

Future studies should evaluate how coordinated in-person assessment, teleconsultation, and remote follow-up can be combined within integrated cough care pathways [126].

Virtual multidisciplinary models have already demonstrated the feasibility of integrating pre-visit symptom assessment and medical record review with coordinated interprofessional evaluation and video-based consultations in patients with chronic cough [127]. More broadly, virtual approaches to chronic cough assessment and management, including specialist teleconsultation, remotely delivered speech-language pathology interventions, and digital follow-up, appear feasible and acceptable, although the current evidence base is limited and heterogeneous [128].

Remote BCST delivery may help address limited access to specialist care. Clinician-delivered telehealth BCST has been associated with symptom improvement in an observational study [129]. A small randomized trial of asynchronous internet-based BCST also reported greater improvement in cough-related quality of life than healthy-lifestyle education [130]. These findings warrant further evaluation of remote treatment delivery but do not establish equivalence to face-to-face BCST or demonstrate the effectiveness of a complete virtual multidisciplinary pathway.

Pediatric studies should determine whether selected components of treatable trait assessment, objective cough phenotyping, digital monitoring, and multidisciplinary integration can be safely and meaningfully adapted within pediatric-specific diagnostic frameworks [95,97,98].

Prospective validation of the proposed framework should begin by assessing feasibility, inter-rater agreement across clinicians and specialties, and reproducibility of mechanistic weighting in clinically stable patients. Longitudinal studies should examine whether changes in assigned mechanistic weights correspond to treatment response and clinically meaningful outcomes.

Subsequent multicenter pragmatic studies should compare the framework with guideline-based usual care, assessing cough-related quality of life, objective cough frequency, and time to effective treatment. Diagnostic efficiency, investigations and specialist visits, medication and patient burden, healthcare utilization, patient satisfaction, and cost-effectiveness should also be evaluated [81,82].

## 11. Conclusions

Chronic cough is increasingly understood as a multidimensional clinical syndrome rather than a symptom confined to a single organ system. Its persistence may reflect interactions among lower-airway inflammation, upper-airway disease, reflux-related sensory activation, laryngeal dysfunction, cough hypersensitivity, sensory-neural dysregulation, behavioral factors, and other patient-specific or modifiable contributors. This complexity helps explain why disease-centered and sequential single-specialty approaches may be insufficient, particularly in patients with refractory or unexplained chronic cough.

The mechanism-based multidisciplinary framework proposed in this review integrates guideline-based disease assessment with characterization of cough hypersensitivity and laryngeal involvement, provisional weighting of identified traits and contributors, targeted multidisciplinary input, individualized intervention, and response-guided reassessment. Rather than simply cataloguing coexisting abnormalities, the framework aims to make therapeutic prioritization explicit and to use discordance between trait response and cough response to refine the working mechanistic interpretation. The proposed weighting categories and nine-step pathway should currently be regarded as an evidence-informed conceptual model rather than a validated diagnostic algorithm or standard of care.

The next challenge in chronic cough is therefore not simply to identify additional causes or develop additional antitussive therapies, but to determine which mechanisms are clinically relevant in which patients and how those mechanisms should be prioritized and reassessed over time. Prospective studies should establish whether this structured approach is reproducible, feasible, and capable of improving patient-important outcomes, diagnostic efficiency, and cost-effectiveness compared with guideline-based usual care. If validated, such an approach may support the transition from fragmented disease-centered management toward more coordinated and individualized chronic cough care.

## Figures and Tables

**Table 1:** Key Concepts Underpinning Contemporary Chronic Cough Management.

Concept	Operational Meaning in Chronic Cough
Chronic cough	Cough lasting more than 8 weeks in adults.
Refractory chronic cough (RCC)	Chronic cough that persists despite adequate evaluation and appropriate treatment of identified causes or treatable traits.
Unexplained chronic cough (UCC)	Chronic cough without an established cause after guideline-based investigations and supervised trials of clinically indicated treatments.
Cough hypersensitivity syndrome (CHS)	A clinical syndrome characterized by heightened cough responsiveness to otherwise innocuous or low-level thermal, chemical, mechanical, or laryngeal stimuli.
Treatable trait	A clinically identifiable and potentially modifiable mechanism or characteristic that contributes to cough generation, amplification, or persistence and can be specifically targeted by therapy.
Modifiable contributor	A potentially reversible factor that may cause, trigger, amplify, or perpetuate cough without necessarily representing a distinct disease or treatable trait.

These concepts are complementary and are not mutually exclusive.

**Table 2:** Complementary Roles of Clinical Specialties in Multidisciplinary Assessment and Management of Chronic Cough.

Specialty	Contribution to Integrated Care	Additional Domains to Consider
Pulmonology	Assess asthma (including cough-variant asthma), NAEB, COPD, bronchiectasis, and ILD; use spirometry, FeNO, and imaging as indicated to characterize lower-airway traits.	Laryngeal dysfunction, cough hypersensitivity, and extrapulmonary contributors.
Otorhinolaryngology	Assess rhinitis/CRS, laryngeal hypersensitivity symptoms, inducible laryngeal obstruction, and phonation-triggered cough; interpret upper-airway and laryngeal findings in the clinical context.	Eosinophilic lower-airway disease, reflux-related contributors, and broader cough hypersensitivity.
Allergy/Immunology	Assess atopy, allergic rhinitis, type 2 inflammation, and eosinophilic airway traits; interpret relevant biomarkers to guide trait-directed therapy.	Laryngeal dysfunction and sensory-neural contributors.
Gastroenterology	Assess acid and non-acid reflux, esophageal dysmotility, and reflux–cough associations; interpret relevant investigations to assess the clinical relevance of findings.	Laryngeal and cough hypersensitivity; competing non-reflux mechanisms.
Neurology	Consult when a coexisting neurological disorder is suspected or relevant neurological signs are present; assess potential contributions to cough or impaired airway protection.	Concurrent airway, laryngeal, and reflux-related contributors.
Speech-Language Pathology	Assess cough control and laryngeal function; provide behavioral cough suppression, breathing retraining, and laryngeal hygiene advice alongside indicated medical treatment.	Inflammatory, structural, and reflux-related disease requiring medical treatment.
Behavioral Medicine/ Psychology	Assess psychological comorbidity, hypervigilance, coping, and symptom-related distress; address behavioral factors affecting symptom burden and cough control.	Structural, inflammatory, and sensory-neural contributors.

Specialty involvement is guided by clinical findings; the roles are complementary and non-exclusive (COPD, chronic obstructive pulmonary disease; CRS, chronic rhinosinusitis; FeNO, fractional exhaled nitric oxide; ILD, interstitial lung disease; NAEB, nonasthmatic eosinophilic bronchitis).

**Table 3:** Representative Mechanistic Patterns and Clinical Contexts Relevant to Chronic Cough Assessment.

Clinical Dimension	Representative Pattern	Clinical Clues	Potential Mechanisms/ Contributors	Assessment Priorities	Treatment Implications
Mechanistic patterns	Lower-Airway– Reflux–Upper- Airway Overlap	Rhinitis/CRS/allergic rhinitis; asthma or eosinophilic airway disease; reflux symptoms; variable triggers	Type 2/eosinophilic inflammation; upper-airway inflammation; reflux-related afferent activation; interacting airway mechanisms	Spirometry; FeNO/blood eosinophils; upper-airway evaluation; reflux assessment when indicated	Combined trait-based therapy; anti-inflammatory treatment; upper-airway management; reflux-directed therapy; address behavioral/environmental factors
	Cough Hypersensitivity- Dominant	RCC/UCC; talking/laughing/odors/cold air/swallowing triggers; urge-to-cough; minimal routine findings	Possible peripheral and central neural sensitization; heightened cough responsiveness; proposed neuroimmune involvement	CHS assessment; LCQ/VAS; objective cough monitoring when available; review coexisting traits/red flags	BCST/SLP; breathing retraining; neuromodulators as appropriate; targeted antitussive therapy where available
	Laryngeal Hypersensitivity- Dominant	Throat irritation, globus, dysphonia; throat clearing; phonation/odor/cold-air triggers; ILO/dysfunctional breathing	Laryngeal sensory-motor dysfunction; laryngeal hypersensitivity; ILO; abnormal laryngeal/respiratory sensorimotor behavior	Flexible laryngoscopy; ILO assessment; SLP evaluation; breathing/voice assessment; evaluate coexisting traits	BCST; breathing retraining/ILO management; laryngeal rehabilitation/voice therapy; treat coexisting airway/reflux disease
Clinical-context patterns	Post-Infectious/ Post-Viral Cough	Persistent cough after respiratory infection; often dry/prolonged; heightened sensitivity after acute illness	Possible post-inflammatory/neuroimmune changes and persistent cough hypersensitivity	Exclude active infection/red flags; assess other causes; evaluate cough hypersensitivity if persistent; impact assessment	Reassurance/observation when appropriate; time-limited trials for coexisting traits; mechanism-directed management
	Older Adult: Frailty, Dysphagia and Airway Protection	Frailty; dysphagia/cough during meals; choking; recurrent pneumonia; comorbidities/polypharmacy	Dysphagia/aspiration; impaired airway protection; reflux/chronic lung disease; neurological disease; altered cough reflex	FEES/VFSS when indicated; aspiration risk; pulmonary evaluation; medication review; frailty assessment	Swallowing-directed management; aspiration prevention; treat contributing respiratory/reflux traits; caution with cough suppression

These non-exclusive patterns are intended to support clinical reasoning and do not constitute a validated classification or molecular endotypes (BCST, behavioral cough suppression therapy; CHS, cough hypersensitivity syndrome; CRS, chronic rhinosinusitis; FEES, flexible endoscopic evaluation of swallowing; FeNO, fractional exhaled nitric oxide; ILO, inducible laryngeal obstruction; LCQ, Leicester Cough Questionnaire; RCC, refractory chronic cough; SLP, speech-language pathology; UCC, unexplained chronic cough; VAS, visual analogue scale; VFSS, videofluoroscopic swallowing study).

**Table 4:** Selected Pharmacological and Non-Pharmacological Therapeutic Options for Refractory and Unexplained Chronic Cough.

Intervention	Pharmacological Target/ Proposed Antitussive Rationale	Evidence	Potential Clinical Role	Key Limitations/ Considerations
Gabapentin	α2δ calcium-channel subunit binding; proposed modulation of neuronal excitability and sensory processing. The precise mechanism underlying clinical antitussive benefit remains uncertain.	Randomized placebo-controlled trial demonstrated improvement in cough-related quality of life in RCC.	Selected patients with RCC/UCC and prominent cough hypersensitivity.	Dizziness, fatigue, somnolence and cognitive effects; variable response; careful patient selection and monitoring required.
Pregabalin	α2δ calcium-channel subunit binding; proposed modulation of neuronal excitability and sensory processing. The precise antitussive mechanism remains incompletely defined.	Randomized trial in combination with speech pathology showed greater improvement in cough severity and cough-related quality of life than speech pathology alone.	Selected refractory patients, particularly when combined with behavioral/speech-language pathology intervention.	Neurological adverse effects and tolerability; evidence for cough is more limited than for gabapentin.
Amitriptyline	Monoamine-transporter interactions and other pharmacological actions; their contribution to antitussive benefit is not established.	Small randomized study in chronic cough attributed to postviral vagal neuropathy suggested benefit.	Limited-evidence option in selected patients after specialist assessment; routine RCC/UCC use is not established.	Limited evidence base; anticholinergic and sedating adverse effects; findings should not be generalized broadly to RCC/UCC.
Paroxetine	Selective serotonin reuptake inhibition; cough-specific mechanism uncertain.	Very limited observational evidence: two responders among seven treated patients in a retrospective RCC cohort.	Reported use in selected patients; insufficient evidence for routine RCC/UCC treatment.	Very small uncontrolled subgroup; efficacy and cough-specific safety remain uncertain.
Flupentixol/ Melitracen	Antipsychotic–tricyclic antidepressant combination; cough-specific mechanism uncertain.	One short-term placebo-controlled RCT in RCC after prior neuromodulator failure.	Preliminary evidence in selected patients; routine use is not established.	Single-center study; brief follow-up; no objective cough monitoring; somnolence.
Low-dose slow-release morphine	Opioid-receptor agonism; modulation of cough-related airway sensations is a proposed rationale, but the precise antitussive mechanism remains incompletely defined.	Placebo-controlled trials support symptomatic benefit; a short responder-enriched crossover trial also demonstrated reduced objective cough frequency.	Selected adults with RCC under specialist supervision.	Constipation, sedation, and other opioid-related risks; individualized monitoring; limited generalizability of responder-enriched evidence.
Gefapixant	P2X3/P2X2/3 receptor antagonism; reduces ATP-mediated activation of vagal sensory afferents. This pharmacological action does not establish the complete mechanism of clinical cough reduction.	COUGH-1 and COUGH-2 phase 3 trials showed statistically significant reductions in 24-hour cough frequency with 45 mg twice daily, with modest overall benefit.	Mechanism-directed pharmacological option where approved/available for adults with RCC/UCC.	Taste-related adverse events, particularly dysgeusia; modest magnitude of benefit; regulatory availability varies.
Camlipixant	Highly selective P2X3 receptor antagonism; intended to reduce ATP-mediated vagal sensory signaling while limiting P2X2/3-related taste disturbance.	Phase 2b SOOTHE showed reductions in objective cough frequency. In phase 3, CALM-1 met the primary endpoint with 50 mg twice daily, whereas CALM-2 did not; 25 mg twice daily did not meet the primary endpoint in either trial, and key secondary endpoints did not meet target thresholds.	Development for RCC discontinued in July 2026.	Inconsistent phase 3 efficacy; aggregate benefit considered insufficient to support further development for RCC.
BCST/ Speech-Language Pathology	Targets modifiable laryngeal, sensorimotor, breathing-related and behavioral components of cough persistence; no single biological mechanism is assumed.	Randomized trials and evidence syntheses support improvements in cough-related quality of life and cough frequency; PSALTI reduced objective cough frequency by 41% versus control at four weeks in a multicenter RCT.	Important non-pharmacological option, particularly with laryngeal hypersensitivity, urge-to-cough, phonation-triggered cough, dysfunctional breathing or impaired voluntary cough suppression.	Requires trained clinicians and patient engagement; access may be limited; response varies according to clinical phenotype and adherence.

Evidence strength and regulatory availability differ across interventions. Pharmacological targets and proposed rationales do not establish the mechanisms of clinical antitussive benefit; inclusion does not imply routine use. Supporting references are provided in the main text (RCC, refractory chronic cough; UCC, unexplained chronic cough; BCST, behavioral cough suppression therapy; SLP, speech-language pathology; RCT, randomized controlled trial; P2X3, P2X purinoceptor 3; P2X2/3, heteromeric P2X2/3 receptor; ATP, adenosine triphosphate; PSALTI, physiotherapy and speech and language therapy intervention).

**Table 5:** Key Research Gaps and Future Study Priorities in Chronic Cough.

Research Gap	Why It Matters	Future Study Design
1. Mechanistic endotypes and clinical weighting of treatable traits	An identified trait does not establish its causal relevance. Mechanistic endotypes require reproducible biological evidence, whereas the proposed clinical weighting categories remain unvalidated.	Prospective mechanistic phenotyping studies testing structured trait-weighting models against conventional diagnostic approaches.
2. Biomarkers of cough hypersensitivity and sensory-neural dysfunction	Diagnosis remains largely symptom-based, and validated diagnostic or predictive neural biomarkers are lacking.	Prospective multimodal biomarker-discovery and validation cohorts integrating biological, physiological, sensory, and digital measures.
3. Standardized criteria for laryngeal hypersensitivity and dysfunction	Diagnostic definitions remain heterogeneous; laryngoscopic findings alone do not establish sensory hypersensitivity.	Multicenter studies integrating standardized laryngoscopy, symptom profiles, provocation measures, and speech-language pathology assessment.
4. Standardization and phenotyping of behavioral cough suppression therapy (BCST)	Intervention protocols and delivery models vary, and predictors of response remain incompletely defined.	Multicenter pragmatic randomized controlled trials with standardized intervention components and phenotype-stratified response analysis.
5. Identification of responder phenotypes for P2X3 antagonists and other sensory-neural therapies	Average treatment effects may conceal substantial heterogeneity in efficacy and tolerability.	Randomized trials stratified by candidate biomarkers or phenotypes, with objective cough outcomes, prespecified interaction analyses, and independent validation of response predictors.
6. Standardization and clinical implementation of objective and AI-assisted cough monitoring	Platforms, recording duration, validation methods, performance metrics, and clinically meaningful thresholds remain insufficiently standardized.	Prospective real-world validation, cross-platform comparison, external AI validation, implementation, and health-economic studies.
7. Clinical effectiveness of integrated multidisciplinary cough care	Multidisciplinary care is increasingly advocated, but comparative evidence for clinical efficiency, outcomes, and cost-effectiveness remains limited.	Prospective comparative studies of integrated multidisciplinary services versus guideline-based usual care.
8. Chronic cough, aging, swallowing dysfunction, and aspiration risk	Cough hypersensitivity must be distinguished from impaired airway protection and aspiration-related mechanisms in vulnerable older adults.	Prospective geriatric cough-swallow cohorts incorporating objective cough and swallowing assessment.
9. Prospective validation of the proposed mechanism-based multidisciplinary framework	The clinical value of structured phenotyping, mechanistic weighting, targeted multidisciplinary integration, and iterative re-phenotyping remains untested.	Prospective multicenter implementation and comparative-effectiveness studies assessing patient outcomes, diagnostic efficiency, healthcare utilization, and cost-effectiveness against guideline-based usual care.

The study designs are illustrative (AI, artificial intelligence; BCST, behavioral cough suppression therapy; P2X3, P2X purinoceptor 3).

## Data Availability

No new data were created or analyzed in this study. Data sharing is not applicable to this article.
